# Pair consensus decoding improves accuracy of neural network basecallers for nanopore sequencing

**DOI:** 10.1186/s13059-020-02255-1

**Published:** 2021-01-19

**Authors:** Jordi Silvestre-Ryan, Ian Holmes

**Affiliations:** Department of Bioengineering, University of California, Berkeley, 94720 USA

## Abstract

We develop a general computational approach for improving the accuracy of basecalling with Oxford Nanopore’s 1D^2^ and related sequencing protocols. Our software PoreOver (https://github.com/jordisr/poreover) finds the consensus of two neural networks by aligning their probability profiles, and is compatible with multiple nanopore basecallers. When applied to the recently-released Bonito basecaller, our method reduces the median sequencing error by more than half.

**Supplementary Information:**

The online version contains supplementary material available at (10.1186/s13059-020-02255-1).

## Main text

Nanopore sequencers, such as the MinION and related devices from Oxford Nanopore Technologies (ONT), allow for direct readout of individual DNA molecules [1]. However, the higher error rate of nanopore sequencing compared to other methods has limited its application in situations where deep coverage is unavailable, such as detection of rare variants or characterization of highly polymorphic samples. In principle, 2X coverage is available even for single duplexes, using ONT’s 1D^2^ protocol or related methods which sequence both strands of the duplex consecutively. In the 1D^2^ protocol, special DNA adapters are used such that after the template DNA strand passes through the pore, its complementary strand very often follows. Combining the readout of both strands should improve accuracy; however, most neural network basecaller architectures are designed to operate on single strands. Here we present a general method for adapting existing basecallers to take advantage of the extra information in paired 1D^2^ reads.

Nanopore sequencing works by threading a single strand of DNA through a protein nanopore embedded in a synthetic membrane. The DNA bases block the pore, perturbing the ionic current flowing through. The current can be measured, and the original sequence of nucleotides recovered computationally. This latter *basecalling* step makes heavy use of machine learning techniques and, increasingly, of neural networks.

Early neural network basecallers (such as DeepNano [2], BasecRAWller [3], and certain ONT-developed basecallers) relied on a preprocessing step that segmented the current measurements into discrete events, corresponding to individual nucleotides passing through the pore. This aspect of basecalling shares similarities with speech recognition, where an audio time series must be segmented and then labeled with phonemes. Inspired by this similarity, later basecallers used Connectionist Temporal Classification (CTC), a method developed for speech recognition, which trains neural networks to do segmenting and classification simultaneously [4]. The community basecaller Chiron [5] successfully applied CTC to nanopore basecalling [6], while ONT incorporated CTC-style models into both production and research basecallers.

**Fig. 1:**
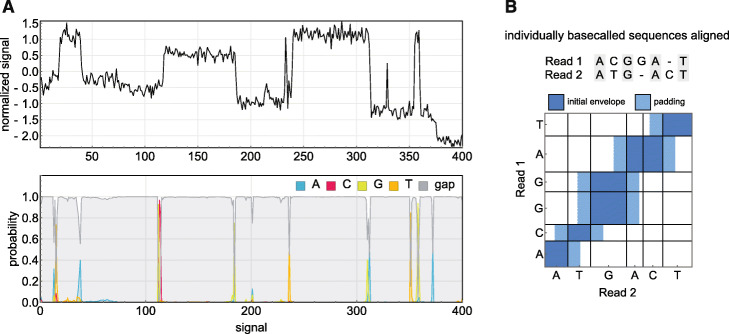
Nanopore basecalling maps signal to sequence. **a** To basecall a single read, the time series of current signal is fed into a neural network basecaller which outputs for each measurement the probabilities of each base plus a blank gap character. This probability profile is then decoded to find the most likely basecalled sequence. **b** To constrain our pair decoding algorithm, each read was basecalled individually and the alignment of the resulting sequences was used to define a region in signal space that banded our 2D beam search

A CTC-trained neural network outputs a probability profile (Fig. 1a) defining a distribution *P*(*ℓ*|*y*) over possible basecalled sequences *ℓ* given the read *y*. By analogy to hidden Markov models, the task of finding the modal sequence of this distribution is termed “decoding”. While perfectly optimal decoding requires an intractably exhaustive search over sequences, heuristic algorithms (such as beam search or Viterbi search) can in practice be used to find reasonably good solutions.

The related task of “consensus decoding” arises when multiple reads {*y*_*n*_} are derived from the same underlying sequence *ℓ*, as is the case for 1D^2^. Basecalling then yields multiple profiles *P*(*ℓ*|*y*_*n*_). Our task is to find the single sequence that maximizes *P*(*ℓ*|{*y*_*n*_}); under a flat prior *P*(*ℓ*) and the assumption that the reads are independent, this will be the sequence that maximizes the product ${\boldsymbol {\prod \nolimits }_{n}} P\left (\ell |y_{n}\right)$, motivating the reframing of this problem as an exercise in profile-profile alignment [7].

To this end we have developed a beam search decoding algorithm for the pair decoding of two reads, making use of a constrained dynamic programming heuristic to speed calculations by focusing on areas of each read which are likely to represent the same sequence (full details provided in Additional file 1). We introduce our basecalling software PoreOver, which implements these decoding algorithms and includes a basic recurrent neural network basecaller (PoreOverNet) for demonstration purposes.

DNA flows through the pore at an average of 450 bases/second; the electrical signal is recorded at 4000 Hz, yielding 9 measurements/base on average. Thus, if a read represents *T* bases, aligning two basecalled reads will take ∼*T*^2^ steps, but aligning the raw signal measurements will take ∼(9*T*)^2^ steps—an 81-fold increase compared to aligning basecalled sequences. To accelerate calculations we constrain our heuristic search to an “alignment envelope” containing the timepoints where the reads are most likely to align [8].

This envelope is estimated by doing a preliminary Viterbi decoding step on each read individually, then aligning the two sequences so obtained. This is faster than beam search, with similar performance (see Additional file 1), and explicitly maps each nucleotide to some range of timepoints. The two decoded sequences are then aligned globally, generating a nucleotide-level mapping between the reads, and (by extension) between the underlying time series. With some additional padding, this guide alignment defines the envelope for our banded 2D beam search (Fig. 1b).

As nanopore reads can vary in length over orders of magnitude, a naive Needleman-Wunsch alignment may involve creating infeasibly large dynamic programming matrices. As a workaround, we use a modified Needleman-Wunsch with a fixed diagonal band. This appears to be sufficient for subsequent pair decoding, though exploiting recent advances in efficient pairwise alignment algorithms (such as [9]), may yield further improvements in accuracy and speed.

We tested our pair decoding algorithm on a sample of 5,000 R9.4 *E. coli* 1D^2^ read pairs (Oxford Nanopore Technologies, personal communication), comprising 10,000 reads in total. Reads were run through a forward pass of our PoreOverNet basecaller to generate softmax probabilities, which were used for subsequent pair decoding.

**Fig. 2:**
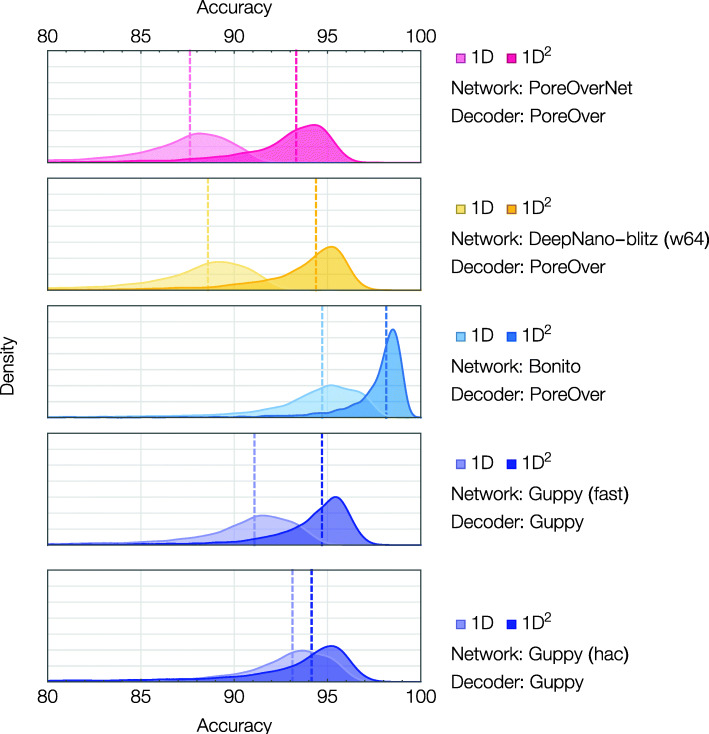
Consensus decoding improves sequencing accuracy. Reads were run through PoreOverNet (magenta), the community basecaller DeepNano-blitz (yellow), and ONT’s Bonito basecaller (blue) to generate softmax probabilities, which were then decoded using our algorithms. Guppy accuracies (in violet) were generated entirely from running the Guppy basecaller and its 1D^2^ basecalling mode without any additional decoding. The Guppy basecaller has the option of two neural network architectures using either smaller (fast) or larger (high accuracy, hac) recurrent layer sizes. DeepNano-blitz was run with its width64 network. The median accuracy is represented by a dashed line

After pair decoding, reads were aligned to the reference *E. coli* genome with Minimap [10] and the read accuracy calculated as (number of matches)/(length of alignment). We find that our banded 2D beam search improves the median accuracy from 87.6% for single reads to 93.2% for 1D^2^ read pairs (Fig. 2), nearly halving the error rate of our PoreOverNet basecaller.

Our software can readily be adapted to work with the output of other neural network basecallers. Application to the recent DeepNano-blitz [11], showed a similar gain in accuracy from consensus decoding. We also applied our algorithm to the ONT basecaller Bonito [12], a research basecaller inspired by recent successes of purely convolutional neural networks in speech recognition, and compared results with Guppy, an earlier ONT basecaller which can make use of 1D^2^. Our consensus method lifts Bonito’s median accuracy from 94.7% to 98.1%, better than halving the median error rate for single read basecalling and surpassing the consensus accuracy of Guppy’s 1D^2^ method (Fig. 2). Unlike Guppy, our code is open source; further, it is modular in design, making it straightforwardly modifiable and re-usable for other basecallers. We thus envision the PoreOver as a consensus decoding tool to be used in concert with a state-of-the-art CTC basecaller such as Bonito. Since initial submission of this paper, the Bonito basecaller now includes an implementation of our pair decoding algorithm (as of version 0.2.0, [12]).

Generalizing beyond a pair of reads, consensus approaches are relevant to *polishing*, the task of refining a draft genome assembly by realigning reads to the draft. There are several approaches to polishing via multi-read consensus: some analyze the raw current signal using a hidden Markov Model [13] or dynamic time warping [14], while others analyze the basecalled sequence using neural networks [15, 16]. To our knowledge none of the neural network methods explicitly use the intermediate basecaller probabilities (instead relying on previously basecalled sequence), while the methods that do use the raw signal do not use neural networks. The pairwise dynamic programming approach we describe could be extended to multiple reads, although the curse of dimensionality (a full dynamic programming alignment of *N* reads takes $\mathcal {O}\left (T^{N}\right)$ steps) would necessitate additional heuristics to narrow down the search space. These could include generalizing alignment envelopes to multiple sequences, or performing a stochastic search. With such heuristics, it should be possible to implement an algorithm to exploit the basecaller probabilities for general, multi-read consensus [7].

## Supplementary Information

**Additional file 1** Supplementary text and figures.

**Additional file 2** Review history.

## Data Availability

Our software PoreOver [17] is available at https://github.com/jordisr/poreoverunder an MIT license. The *E. coli* 1D^2^ reads used to test our pair decoding algorithm were generated by Oxford Nanopore Technologies and are available at [18].
