# Supplementary material for: Pair consensus decoding improves accuracy of neural network basecallers for nanopore sequencing
Source: Genome Biol. 2021 Jan 19;22:38. doi: 10.1186/s13059-020-02255-1 (PMC7814537; doi:10.1186/s13059-020-02255-1)
Supplement: Supplementary file 1 — Additional file 1 Supplementary text and figures. [file 13059_2020_2255_MOESM1_ESM.pdf]

Supplementary Information

Basecalling with connectionist temporal classification

Under CTC, the output of the neural network defines a probability distribution over possible labelings of the input, a "labeling" in this case representing the DNA sequence that passed through the pore. CTC uses a differentiable loss function calculated with dynamic programming to calculate the probability of a given labeling. Using gradient descent, the network is trained to maximize the probability of the correct sequence.

The final softmax layer of the neural network outputs y, a $5 \times T$ matrix that specifies the probability of emitting each base plus a gap character at each step of the input. Let $y(t, c)$ be the probability of the character $c \in \mathcal{G}$ at time t, with $\mathcal{G} = \mathcal{L} \cup \{\epsilon\}$, where characters in $\mathcal{L} = \{A, C, G, T\}$ represent bases passing through the pore and ϵ is a gap or blank character representing no change in the pore. The neural network output can thus be interpreted as a linear hidden Markov model [1], with the softmax probabilities corresponding to emission probabilities in this HMM. The probability of a given path through this profile $\pi \in \mathcal{G}^T$ is just the product of the individual probabilities

$$P(\pi|y) = \prod_{t=1}^{T} y(\pi_t, t)$$

Under this model, sequences longer than T have zero probability.

Each gapped path π can be mapped to an ungapped label sequence ℓ by a function $\mathbf{B} : \mathcal{G}^* \to \mathcal{L}^*$, which simply removes the gap characters. This is a simplifed version of the path-to-label mapping used by the canonical CTC model [2]; unlike the original, our version does not merge repeated label characters (so the path `A-CCG--T` would result in the label `ACCGT` rather than `ACGT`). For a given label sequence ℓ, we want to find the probability that ℓ was emitted by y, $P(\ell|y)$. The probability of a label sequence is the sum of probabilities of all paths consistent with it, essentially marginalizing over all possible positions of gaps:

$$P(\ell|y) = \sum_{\pi:\mathbf{B}(\pi)=\ell} P(\pi|y)$$

This probability can be efficiently computed by dynamic programming with a Forward algorithm[3]. We define the Forward probability of a label as $\alpha(t, s)$, the probability that the first s characters of ℓ having been emitted by position t of the underlying HMM. The core recursion is,

$$\alpha(t, s) = y(t, \ell_s)\alpha(t-1, s-1) + y(t, \epsilon)\alpha(t-1, s)$$

terminated by the base case $\alpha(0, 0) = 1$. From this matrix we can easily read out the probability of the full sequence, $P(\ell|y) = \alpha(T, |\ell|)$.

Decoding the basecaller output

For basecalling we want to find the best label, $\hat{\ell}$,

$$\hat{\ell} = \text{argmax}_{\ell} P(\ell|y)$$

Borrowing HMM terminology, this task is referred to as decoding. While there is not an efficient general algorithm for this optimization, various heuristic search algorithms can be used to find high probability sequences. Here we focus on two approximate methods, finding the (1) Viterbi best path, and (2) a beam search.

While finding the best label is intractable, a simpler approach is to instead find the single best path:

$$\hat{\pi} = \text{argmax}_{\pi} P(\pi|y)$$

Thus, the Viterbi solution is $\ell_{\text{Viterbi}} = \mathbf{B}(\hat{\pi})$. In our CTC model, this is equivalent to taking the argmax of each output in the time series and removing the gaps.

An alternative heuristic search method is beam search, which has been used extensively in the decoding of neural networks, including CTC models[4]. Beam search iterates through the output y, keeping a fixed-size list (or 'beam') of the best solutions. The size of this list is parameter termed the beam width, and represented with W. The algorithm is the following: for each iteration t in $\{1..T\}$, update its probability at time t using the forward recursions. Next, extend each label in the beam by each character in the alphabet $\{A, C, G, T\}$ and calculate the corresponding forward probabilities. Finally, prune the beam down to the W labels with the highest probabilities. By increasing the beam width W, more solutions are tracked at every iteration.

Pair decoding

Assuming the independence of each read, and a flat prior over labels, the best label is given by

$$\hat{\ell} = \text{argmax}_{\ell} P(\ell|y_1, y_2) = \text{argmax}_{\ell} P(\ell|y_1) P(\ell|y_2)$$

We extend our beam search to work in two dimensions over the $T_1 \times T_2$ space of both reads. Much as in each iteration of a standard beam search, the extensions of each sequence are added to the beam and the score of each sequence is updated. Finally, the beam is pruned down to the top W hits with the highest scores, where W is the beam width. We present below two variations of the algorithm.

In the first, we iterate over $t_t \in 1..T_1$, and at each step update the forward probabilities for labels in the beam at t_1 and for $t_2 \in 1..T_2$. Each label in the beam is assigned a score equal to the forward probability from read 1 at t_1 times the maximum forward probability from read 2 in the range $1..T_2$.

$$\text{score}(\ell) = \alpha_1(t_1, |\ell|) + \max_{t \in 1..T_2} \alpha_2(t, |\ell|) \tag{1}$$

We also tested a second symmetric variation, where each iteration of the search considers both a column $(1..T_1)$ and a row $(1..T_2)$ of signal space, and the beam scores are weighted by the respective maximum forward probabilities in those ranges. At each iteration both t_1 and t_2 are incremented by one.

$$\text{score}(\ell) = \max_{t \in 1..T_1} \alpha_1(t, |\ell|) + \max_{t \in 1..T_2} \alpha_2(t, |\ell|) \tag{2}$$

Both of these strategies are implemented in PoreOver and yield similar accuracies on our test data. The second method reduces the number of beam update steps and runs comparatively faster when adapted to use the alignment envelope, and so was used for the accuracy benchmarks in Fig 2 (and is the default setting of PoreOver).

Because the complementary strand passes through the pore with high probability but not every read generates a second strand, there is an additional step during sequencing to determine whether two reads are complementary strands or not. For this work we just relied on the determination of the Guppy basecaller. While one could in theory train two RNNs to operate on the forward and reverse strands and then run consensus on those two probabilities, here we adopt a simpler approach by taking the "reverse complement" of the softmax probabilities, for example:

$$
\begin{array}{c} t= \\ A \\ C \\ G \\ T \\ \epsilon \end{array}
\begin{array}{c} \begin{array}{ccc} 1 & 2 & 3 \end{array} \\ \begin{bmatrix} 0.5 & 0 & 0.5 \\ 0 & 0.6 & 0.5 \\ 0 & 0.2 & 0 \\ 0 & 0 & 0 \\ 0.5 & 0.2 & 0 \end{bmatrix} \end{array}
\xrightarrow{\text{reverse complement}}
\begin{array}{c} t= \\ A \\ C \\ G \\ T \\ \epsilon \end{array}
\begin{array}{c} \begin{array}{ccc} 1 & 2 & 3 \end{array} \\ \begin{bmatrix} 0 & 0 & 0 \\ 0 & 0.2 & 0 \\ 0.5 & 0.6 & 0 \\ 0.5 & 0 & 0.5 \\ 0 & 0.2 & 0.5 \end{bmatrix} \end{array}
$$

For Bonito decoding, we modified the basecaller (version 0.2.2) to save softmax probabilities and adapted our decoding algorithm to work with a CTC model that merges repeated characters.

Pair decoding can be trivially parallelized over multiple reads for a large speedup. For our Bonito experiment, decoding the 5,000 read pair test set using 20 parallel processes on an Intel i9-9820X workstation took 17:38 minutes, yielding an average decoding speed of 1,628 consensus bp/s/thread.

Our pair decoding algorithm makes use of a very narrow alignment envelope defined in signal space using a sequence alignment. Given a match state in the sequence alignment, and the default padding of 5 on either side, we would expect the average size to be 9+5+5 =19. Empirically, we see a mean envelope width of 22, not far off this estimate. For two average reads of 10kb and 90,000 signals, this envelope would constrain the search to just 0.024% of the entire matrix, an efficiency which helps justify our use of a pairwise sequence alignment step.

The quality of the consensus sequence tends to depend on the quality of this sequence alignment, and indeed the main failure mode appears to be cases where the two $1D^2$ reads are unalignable. As the algorithm assumes sequences will be globally alignable, any sequences with a significant length mismatch are skipped by PoreOver. Of the remaining reads, a small fraction have poor pairwise alignments that results in poor consensus sequences (i.e. consensus accuracy lower than the mean 1D accuracy of the two reads), sometimes due to one read being of significantly worse quality and bringing the overall average down. Altogether, including the sequences that are skipped, these failure cases make up $< 2\%$ of the our test data.

Pair decoding and genome assembly

We sought to investigate whether the accuracy improvement from consensus decoding would carry through downstream assembly and polishing. To do this we created two assemblies from our set of 5,000 paired reads. In the first, we ignored the pairing information and treated each read independently, basecalling with the Bonito network and decoding with the Viterbi algorithm. For the second assembly, we basecalled each read with Bonito but then ran PoreOver to generate consensus sequences for each pair. The resulting sets of sequences were assembled with miniasm [5] and then polished for four rounds with Racon [6]. The resulting contigs were compared against the reference and the results are shown in Figure S3. We find that while the unpolished assembly of $1D^2$ reads is significantly more accurate, this difference is reduced after several rounds of polishing. Thus, this highlights that the main use of pair decoding is for maximizing single-molecule accuracy, and has only a marginal benefit when used in an assembly+polishing workflow.

Neural network architecture and training

Our example basecalling network, PoreOverNet, consists of a single convolutional layer followed by three bidirectional GRU[7] layers (Figure S1). Output is passed through a softmax function to yield probabilities for each nucleotide plus a gap character, $\{A, C, G, T, \epsilon\}$. Under this model, the gap character represents no change in the pore, and so the output probability trace consists of peaks of probability as each nucleotide passes through the pore, followed by stretches with high gap probability (Figure 1A).

Given that training organism influences the generalizability of the network[8], we sought to include a broad taxonomic diversity. A training set of R9.4 reads was assembled from a sample of 10,000 human reads from the nanopore whole genome sequencing consortium[9] and 10,000 microbial reads from the Zymo mock community[10] spanning 8 bacterial and 2 yeast species.

Reads were re-bascalled with the Guppy basecaller (version 3.2.4) and mapped back to reference genomes. Tombo[1] was used to re-align the raw signal to the reference sequence and correct previous basecalling errors. These corrected reads were split into chunks of 1000 measurements and used as the training set. A fraction of the data was held out as a test set and used to evaluate the performance of our model during training. The neural network was trained for ten epochs using the Adam optimizer [11] (learning rate of 0.0005) to minimize CTC loss.

Evaluating decoding algorithms for single read basecalling

In addition to the read pair decoding presented in the main text, we also compare single read decoding algorithms using both our own trained basecalling network (Figure S1), as well as ONT's basecaller Guppy, which implements a variant of CTC called "flip-flop". The same test set of 10,000 reads was used as in the previous benchmark, though the pairing information was ignored and reads were treated as standard 1D reads.

[1] `https://github.com/nanoporetech/tombo`

Simplified CTC model

Test reads were split into chunks of 1000 measurements, batched together, and fed through PoreOverNet to yield the softmax probabilities. Decoding was then done with both (1) Viterbi best path and (2) a beam search (Figure S2A). The use of beam search over Viterbi yielded a very slight improvement in median accuracy from 87.6% to 88.1%. Interestingly, the beam width had little effect on the overall accuracy, with $W = 50$ yielding nearly identical results. While beam search does yield a slight improvement over Viterbi decoding, it comes at a disproportionately greater computational cost.

Flip-flop CTC model

While the earliest nanopore basecallers focused on the task of predicting a sequence of k-mers [12], the production basecaller Guppy along with the research basecaller Flappie introduced a character level, CTC-style model known as "flip-flop". The flip-flop model is an adaptation of CTC for the purpose of better calling homopolymers, a known error mode in nanopore sequencing.

The flip-flop model does not use gaps, as in the standard CTC model but instead has two sets of "flip" (+) and "flop" (-) states, with transitions within flip and flop states only emitting a blank character ϵ, $y(c,t)$ with $c \in \{A^+, C^+, G^+, T^+, A^-, C^-, G^-, T^-\}$. Furthermore, transitions from flip to flop states are only allowed between the same nucleotide (e.g. $A^- \rightarrow A^+$). Internally, the basecaller generates a transition matrix for each time step and then runs a Viterbi decoding to generate the final basecalled sequence. The marginalized version of these transition probabilities are stored in FAST5 files, allowing us to use our own decoding algorithms on the flip-flop probabilities. However, we need to adapt the calculation of the forward probabilities to take into account the flip and flop states:

$$\begin{aligned}\alpha^+(t,s) =& \alpha^+(t-1,s) \cdot y(t,\ell_s) \\ &+ \begin{cases} \alpha^-(t-1,s-1) \cdot y(t,\ell_s) & \text{if } \ell_s = \ell_{s-1} \\ (\alpha^+(t-1,s-1) + \alpha^-(t-1,s-1)) \cdot y(t,\ell_s) & \text{otherwise} \end{cases}\end{aligned}$$

$$\begin{aligned}\alpha^-(t,s) =& \alpha^-(t-1,s) \cdot y(t,\ell_s+4) \\ &+ \begin{cases} \alpha^+(t-1,s-1) \cdot y(t,\ell_s+4) & \text{if } \ell_s = \ell_{s-1} \\ 0 & \text{otherwise} \end{cases}\end{aligned}$$

Results from running on the same set of 10,000 reads are shown in Figure S2B. Surprisingly for the flip-flop model, the Viterbi algorithm actually outperforms the beam search on this data. The Viterbi decoding yielded a median accuracy of 90.9% while beam search decoding yielded a median accuracy of 88.9%. Despite the sequences returned by the beam search having higher probabilities, they tend to be less accurate when aligned to the reference genome. Likely because of this, our pair decoding algorithm, which is a form of beam search, does not work well with flip-flop basecalling.

For some reason, it appears that the more probable sequences aren't necessarily more accurate. While it is unclear why this was the case with the flip-flop model but not our simplified CTC model, it could be symptomatic of the way these models are trained and evaluated. Much as in several natural language processing tasks, there is a mismatch between the maximum likelihood objective used in training, the CTC loss, and the actual metric used for evaluation, the edit distance or alignment accuracy between the predicted and true labelings. While this metric is discrete and non-differentiable, there has been some success in speech recognition using techniques from reinforcement learning to approximate this gradient and optimize the edit distance directly[4]. Applying policy gradient style approaches that maximize the reward function (in this case alignment accuracy) to CTC basecalling could be an avenue for future research.

In addition to the "flip-flop" model available in the production basecaller Guppy, ONT have also recently introduced another basecalling paradigm known as "run-length encoding, which is implemented in the research basecaller Runnie. Under the run-length encoding model, the neural network outputs the best nucleotide as well as parameters of a discrete Weibull distribution which characterizes the length of the repeat. While this makes single read decoding trivial (by predicting the mode of the parameterized distribution), the 2D beam search described could be adapted to work for run length encoded output. Indeed, one of the strengths of beam search is the ease with which it can be adapted (e.g. to use a language model in speech recognition).

References

[1] Silvestre-Ryan, J. & Holmes, I. Consensus Decoding of Recurrent Neural Network Basecallers. In Jansson, J., Martín-Vide, C. & Vega-Rodríguez, M. A. (eds.) *Algorithms for Computational Biology*, 128–139 (Springer International Publishing, Cham, 2018).

[2] Graves, A., Fernández, S., Gomez, F. & Schmidhuber, J. Connectionist Temporal Classification: Labelling Unsegmented Sequence Data with Recurrent Neural Networks. In *Proceedings of the 23rd International Conference on Machine Learning*, ICML '06, 369–376. ACM (ACM, New York, NY, USA, 2006). URL `http://doi.acm.org/10.1145/1143844.1143891`.

[3] Durbin, R., Eddy, S. R., Krogh, A. & Mitchison, G. *Biological sequence analysis: probabilistic models of proteins and nucleic acids* (Cambridge university press, 1998).

[4] Graves, A. & Jaitly, N. Towards end-to-end speech recognition with recurrent neural networks. In *International Conference on Machine Learning*, 1764–1772 (2014).

[5] Li, H. Minimap and miniasm: Fast mapping and de novo assembly for noisy long sequences. *Bioinformatics* **32**, 2103–2110 (2016). `1512.01801`.

[6] Vaser, R., Sović, I., Nagarajan, N. & Šikić, M. Fast and accurate de novo genome assembly from long uncorrected reads. *Genome Research* **27**, 737–746 (2017).

[7] Cho, K. *et al.* Learning phrase representations using RNN encoder-decoder for statistical machine translation. *arXiv preprint arXiv:1406.1078* (2014).

[8] Stoiber, M. & Brown, J. BasecRAWller: Streaming Nanopore Basecalling Directly from Raw Signal. *bioRxiv* 133058 (2017).

[9] Jain, M. *et al.* Nanopore sequencing and assembly of a human genome with ultra-long reads. *Nature Biotechnology* **36**, 338 (2018). URL `https://doi.org/10.1038/nbt.4060http://10.0.4.14/nbt.4060https://www.nature.com/articles/nbt.4060{#}supplementary-information`.

[10] Nicholls, S. M., Quick, J. C., Tang, S. & Loman, N. J. Ultra-deep, long-read nanopore sequencing of mock microbial community standards. *GigaScience* **8** (2019). URL `https://doi.org/10.1093/gigascience/giz043`.

[11] Kingma, D. P. & Ba, J. Adam: A Method for Stochastic Optimization 1–15 (2014). URL `http://arxiv.org/abs/1412.6980`. `1412.6980`.

[12] Rang, F. J., Kloosterman, W. P. & de Ridder, J. From squiggle to basepair: computational approaches for improving nanopore sequencing read accuracy. *Genome Biology* **19**, 90 (2018).

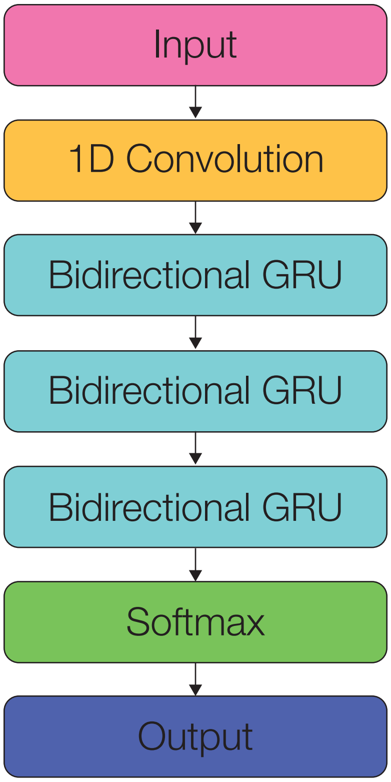

Figure S1: Architecture of PoreOverNet. Input is processed with a single convolutional layer (256 filters, kernel of length 9, stride of 1) followed by three stacked bidirectional GRU layers (128 units each). The model has 893,189 parameters in total. The softmax output is fed into a CTC loss function, and minimized during training.

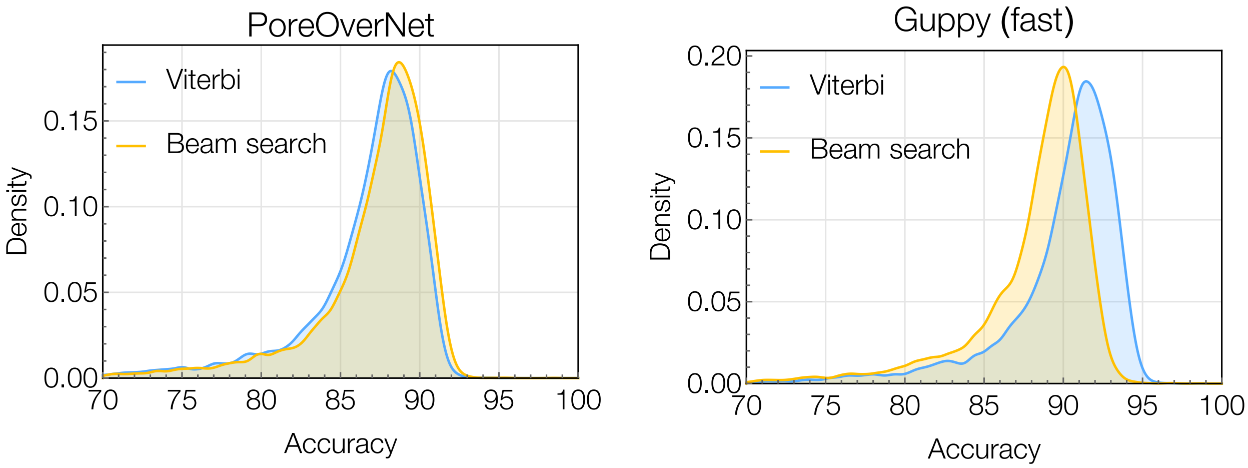

Figure S2: (A) Comparison of single read decoding algorithms using output from our network PoreOverNet, which implements a simplified CTC model that does not merge repeated characters. (B) Single read decoding algorithms run on output of the Guppy basecaller, which implements a variation of CTC for calling homopolymers called “flip-flop”. In this case the beam search surprisingly returned lower accuracy basecalls than Viterbi decoding. A beam width of 10 was used for both plots.

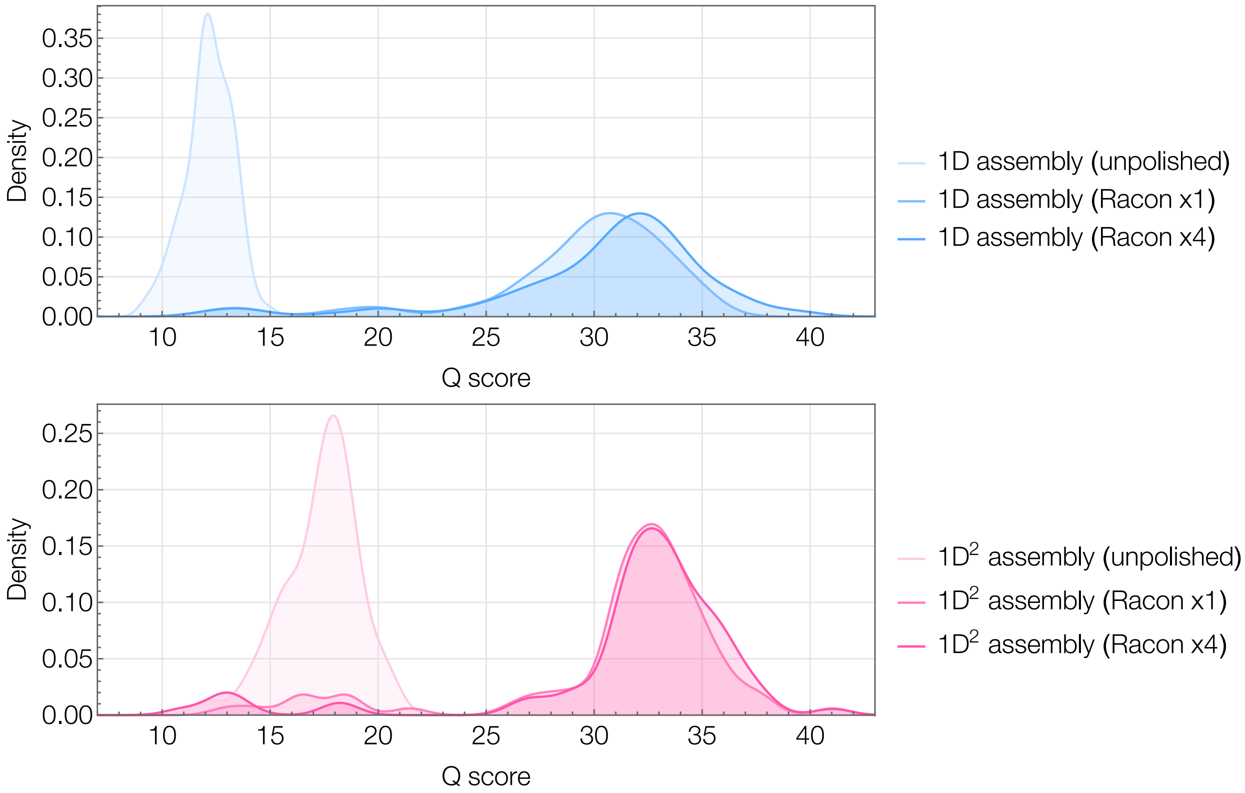

Figure S3: Accuracy of assemblies generated with and without consensus decoding of read pairs. After initial assembly with miniasm, assemblies were polished for several rounds with Racon. The Q-score is defined as $-10\log(\text{error rate})$.
