# Supplementary material for: Pair consensus decoding improves accuracy of neural network basecallers for nanopore sequencing
Source: Genome Biol. 2021 Jan 19;22:38. doi: 10.1186/s13059-020-02255-1 (PMC7814537; doi:10.1186/s13059-020-02255-1)
Supplement: Supplementary file 2 — Additional file 2 Review history. [file 13059_2020_2255_MOESM2_ESM.docx]

Review History

**First round of review**

**Reviewer 1**

**Are you able to assess all statistics in the manuscript, including the appropriateness of statistical tests used?**

No, I do not feel adequately qualified to assess the statistics.

**Comments to author:**

The authors present PoreOver, an approach to improve the basecalling accuracy while sequencing both strands of a DNA duplex (1D^2). Both the improvements discussed here and incrementally achieved internally at ONT show that basecalling is not yet a solved problem and more innovations remain possible to further mature the field. My understanding of the mathematical concepts underlying the machine learning approaches described here is insufficient to evaluate the theoretical part of this manuscript and my comments are therefore rather from an applied perspective. While technical, the manuscript is well written and concise. Please find my minor questions and suggestions below.

Sincerely,

Wouter De Coster

-It is not clear to me why the PoreOver consensus decoding was not used on the Guppy (fast/hac) basecalls. Would this be possible or is the required data not available from Guppy?

-Could the authors comment on why the Guppy 1D2 decoder is worse for hac basecalls than for fast basecalls? To me, this comes as a surprise.

-The improvements in single-read accuracy are encouraging, and the authors mention the intractable possibility to do polishing with this approach without further heuristics. I do think it would be informative to discuss the improvements in assembly/consensus accuracy across multiple 'PoreOver-resolved' 1D^2 reads without necessarily using the neural network information at this stage and while taking into account, if I recall correctly, the lower yield obtained with 1D^2 library preparation compared to 1D preparations.

**Reviewer 2**

**Are you able to assess all statistics in the manuscript, including the appropriateness of statistical tests used?**

Yes, and I have assessed the statistics in my report.

**Comments to author:**

Herein, the authors present a novel algorithm for establishing a consensus between basecalled complementary strands which improves the accuracy of Oxford Nanopore 1D^2 basecalling. There are several points that I would like the authors to address.

Typo in Page 3, Line 15: uses -> use

For the neural network architecture in Figure S1, the number of neurons, kernel size, and activations should be specified along with the total number of parameters in the model.

The presentation of basecalling via PoreOver and pair consensus decoding via PoreOver is confusing at times. Page 2, Lines 54-61 seem to suggest that a typical workflow would be using PoreOver basecalling followed by pair consensus decoding. However, as the authors state (Page 3, Line 17) and show (Fig. 2) that both Bonito and Guppy outperform PoreOver basecalling on single reads, and Fig. 2 shows that the difference in accuracy is considerable. Hence, it is not clear to me what advantages, if any, doing the basecalling with PoreOver might have. In addition, if we are to judge PoreOver as a basecaller, then we need more information about practical considerations like speed and demultiplexing. In my opinion, it should be clarified that the main use of PoreOver is improving Bonito where it has a clear use and outperforms Guppy (as the authors say on Page 3, Lines 13-24).

Can the 1D^2 consensus algorithm presented here fail? If so, how often and why?

The authors specify that they use Needleman-Wunsch to do a global sequence alignment between the read and its complement, which they have implemented in poreover/align/align.pyx. If a read is N bases long, then such a global alignment with Needleman-Wunsch is O(N^2). Oxford Nanopore is known for long reads, and for a 100 kb read this is 10 billion matrix entries to fill in. Even a more typical 10 kb read requires 100 million entries, and from Lines 38-45 in align.pyx, it looks like the software is indeed filling in the entire matrix without any kind of simplifying heuristic. Is this scalable with the distribution of read lengths typically seen with Oxford Nanopore? What kind of runtimes and memory usage to you see?

For a read length of N, the heuristic in Fig. 1B constrains the beam search to N ~9 x ~9 "local" alignments in the best case, but no information is given about the average case. Please comment on the average PoreOver padded envelope sizes compared with established alignment heuristics like the Sakoe-Chiba band, the Itakura parallelogram, and an adaptive banded alignment (Suzuki and Kasahara, BMC Bioinformatics 2018) which do not need the Needleman-Wunch overhead.

For reproducibility, the authors should specify which commit of the software was used for the analysis here or tag a release and specify the tag.

Software issues (as of commit 00ed544):

-	Running "poreover train --data poreover/examples/training/toy --training_steps 100" throws "poreover: error: unrecognized arguments: --training_steps 100". I am guessing at some point this argument was changed to epochs, but this should be updated.

-	poreover/examples/training/toy is not a directory but rather poreover/examples/training contains two gzipped files, which does not seem to be the correct input.

-	Running "poreover call --fast5 poreover/examples/read.fast5 --model run-0 --out test.fasta" throws an error as expected because the model training failed, but the error thrown is "poreover call: error: the following arguments are required: --weights" so it does not look like this command is providing a required argument.

**Reviewer 3**

**Are you able to assess all statistics in the manuscript, including the appropriateness of statistical tests used?**

There are no statistics in the manuscript.

**Comments to author:**

The author developed an algorithm and a package aimed at improving the accuracy of nanopore

sequencing methods when making use of the 1D2 protocol, protocol that sequence both strands of a

DNA duplex sequentially. The originality of the method is the use on an intermediary results of

available basecallers : the probability over the possible bases assigned at each current step for both

complementary sequences. By using this information, through the use of a beam search algorithm

on both strands, the authors manage to improve the accuracy of the last basecaller from nanopore,

bonito, from an accuracy of 93.3 to 97.7 %

Major critique :

1) No clear superiority of their algorithm was demonstrated. A possible comparison could have been

to compare the present algorithm (using guppy output probability) to the 1D2 algorithm in guppy.

2) Two month ago pair decoding was added to bonito basecaller. A comparison between poreover

and bonito should be run. Without it, it is very difficult to assess the added value of the current

method.

3) Before a release of the package, a detailed step by step method explicitly showing how to use

poreover should be added, as for now, by reading the documentation it is not explained how to

generate examples/1d2/read1.npy and examples/1d2/read2.npy which are

required to use poreover in pair-decoding. Also it is mentioned that it is necessary to recompile

bonito to use the method developed by the authors, but there is no information about this on the

readme.

Minor :

3) The author should mention the running time of their algorithm, as nanopore sequencers generate

large volume of data, and this information could be useful in the decision to use or not the current

algorithm.

4) What is the effect of the bandwidth W on the final accuracy?

5) A more symmetric approach could have been use in the pair decoding algorithm: Here, if I

understood correctly, at t1 the first strand is used to select the top W sequences , and the second

strand is used, at t1, from t2=0 to T to improve the accuracy to the global forward probability. I call

this probability P(0→t1,t2=0→T2).

A more symmetric approach could have been used with the exact same algorithm but t1 and t2 are

increased from one at each time, and the top W sequence on both P(0→t1,t2=0→T2) and

P(t1=0→T1,0→t2) are selected.

**Authors Response**

**Point-by-point responses to the reviewers’ comments:**

Reviewer #1: The authors present PoreOver, an approach to improve the basecalling accuracy while sequencing both strands of a DNA duplex (1D^2). Both the improvements discussed here and incrementally achieved internally at ONT show that basecalling is not yet a solved problem and more innovations remain possible to further mature the field. My understanding of the mathematical concepts underlying the machine learning approaches described here is insufficient to evaluate the theoretical part of this manuscript and my comments are therefore rather from an applied perspective. While technical, the manuscript is well written and concise. Please find my minor questions and suggestions below.

Sincerely,

Wouter De Coster

-It is not clear to me why the PoreOver consensus decoding was not used on the Guppy (fast/hac) basecalls. Would this be possible or is the required data not available from Guppy?

*Guppy uses a custom variant of CTC loss developed by Oxford Nanopore known as “flip-flop”, which is different from the CTC models used by both Bonito and PoreOver in that it relies on hidden state structure to model length distributions of basecalled regions. As such it appears to rely heavily on the use of the Viterbi algorithm (which reports the most likely hidden state structure), and does not seem to be amenable to the “beam search” marginalization technique we use (which sums out this hidden state structure). Indeed, even in the 1D case where we are not trying to do any consensus decoding, beam search algorithms do not seem to improve the performance of Guppy (Fig S2). In this respect Guppy’s behavior appears to be different from every basecaller we’ve tested, and different from prior reports in the literature. Despite extensive experiments, we have not yet found a more deeply intuitive explanation for this phenomenon, beyond that outlined above; nor have we found a satisfactory workaround that allows us to use beam search with Guppy. Although we cannot explain exactly why 1D beam search doesn’t work well with Guppy, beyond these general arguments about the fragility of the flip-flop technique, we propose that Guppy’s incompatibility with 1D beam search is the best explanation for why 1D^2-consensus beam search also fails to improve the performance of Guppy. We note that all other basecallers tested, including PoreOverNet, Bonito, and the recently released DeepNano-blitz (none of which rely on the idiosyncratic “flip-flop” trick used by Guppy), are improved by our consensus decoding approach, so we feel our algorithm is still quite general.*

-Could the authors comment on why the Guppy 1D2 decoder is worse for hac basecalls than for fast basecalls? To me, this comes as a surprise.

*This discrepancy was surprising to us as well. Unfortunately, as the Guppy 1D2 algorithm is closed source, it is difficult to diagnose the cause of this result.*

-The improvements in single-read accuracy are encouraging, and the authors mention the intractable possibility to do polishing with this approach without further heuristics. I do think it would be informative to discuss the improvements in assembly/consensus accuracy across multiple 'PoreOver-resolved' 1D^2 reads without necessarily using the neural network information at this stage and while taking into account, if I recall correctly, the lower yield obtained with 1D^2 library preparation compared to 1D preparations.

*Thank you for this comment. We have set up a small test to explore 1D2 decoding and assembly accuracy (please see SI and Fig S3). We find that while the initial assembly from pair decoded 1D2 reads is more accurate than the assembly from the same reads without pair decoding, this difference largely disappears after several rounds of polishing with Racon. Thus, our method adds the most value to single molecule accuracy, and has relatively less benefit to an assembly+polishing pipeline where depth of sequencing coverage effectively provides its own form of consensus.*

Reviewer #2: Herein, the authors present a novel algorithm for establishing a consensus between basecalled complementary strands which improves the accuracy of Oxford Nanopore 1D^2 basecalling. There are several points that I would like the authors to address.

Typo in Page 3, Line 15: uses -> use

*The typo has been fixed.*

For the neural network architecture in Figure S1, the number of neurons, kernel size, and activations should be specified along with the total number of parameters in the model.

*A more detailed description of the neural network layers and parameters has been added to the legend of Figure S1.*

The presentation of basecalling via PoreOver and pair consensus decoding via PoreOver is confusing at times. Page 2, Lines 54-61 seem to suggest that a typical workflow would be using PoreOver basecalling followed by pair consensus decoding. However, as the authors state (Page 3, Line 17) and show (Fig. 2) that both Bonito and Guppy outperform PoreOver basecalling on single reads, and Fig. 2 shows that the difference in accuracy is considerable. Hence, it is not clear to me what advantages, if any, doing the basecalling with PoreOver might have. In addition, if we are to judge PoreOver as a basecaller, then we need more information about practical considerations like speed and demultiplexing. In my opinion, it should be clarified that the main use of PoreOver is improving Bonito where it has a clear use and outperforms Guppy (as the authors say on Page 3, Lines 13-24).

*Thank you for this comment. The paragraph in question (pg. 2 ln. 54-61) references the pair decoding workflow regardless of the basecaller used (this has now been clarified in the text). Even if Bonito is used as the basecaller, the first step of pair decoding involves a Viterbi decoding of the Bonito basecaller probabilities, as a fast way of generating a sequence-to-signal mapping.*

*We have renamed our neural network PoreOverNet, to help differentiate it from our decoding implementations. We have also updated the text to further clarify that the best use of our software is as a pair decoder for the output of Bonito.*

Can the 1D^2 consensus algorithm presented here fail? If so, how often and why?

*Yes, the main failure mode appears to be cases where the two halves of the 1D2 read are unalignable. The quality of the consensus sequence tends to depend on the quality of the sequence alignment used to construct the alignment envelope. It is assumed that the sequences will be globally alignable, so any sequences with a significant length mismatch are skipped. Of the remaining reads, a small fraction of them will have a poor pairwise alignment that results in a poor consensus sequence (i.e. consensus accuracy lower than the mean 1D accuracy of the two reads), sometimes due to one read being of significantly worse quality and bringing the overall average down. Altogether, including the sequences that are skipped, these failure cases make up <2% of the our test data.*

The authors specify that they use Needleman-Wunsch to do a global sequence alignment between the read and its complement, which they have implemented in poreover/align/align.pyx. If a read is N bases long, then such a global alignment with Needleman-Wunsch is O(N^2). Oxford Nanopore is known for long reads, and for a 100 kb read this is 10 billion matrix entries to fill in. Even a more typical 10 kb read requires 100 million entries, and from Lines 38-45 in align.pyx, it looks like the software is indeed filling in the entire matrix without any kind of simplifying heuristic. Is this scalable with the distribution of read lengths typically seen with Oxford Nanopore? What kind of runtimes and memory usage to you see?

*Most of the reads in our test set are around 10kb, and the full Needleman-Wunsch alignment is quite tractable (and not the rate-limiting step in consensus decoding). However, a more efficient algorithm would be better for increased generalizability. We have switched to a fixed-band Needleman-Wunsch algorithm to do the initial pairwise sequence alignment. PoreOver defaults to a 1kb band for this alignment, which should scale favorably even for 100kb reads. We have added a note to the manuscript expanding on the choice of pairwise alignment algorithm.*

*We have additionally added a note on pair decoding runtime to the SI.*

For a read length of N, the heuristic in Fig. 1B constrains the beam search to N ~9 x ~9 "local" alignments in the best case, but no information is given about the average case. Please comment on the average PoreOver padded envelope sizes compared with established alignment heuristics like the Sakoe-Chiba band, the Itakura parallelogram, and an adaptive banded alignment (Suzuki and Kasahara, BMC Bioinformatics 2018) which do not need the Needleman-Wunch overhead.

*Our pair decoding algorithm makes use of a very narrow alignment envelope defined in signal space using a sequence alignment. Given a match state in the sequence alignment, and the default padding of 5 on either side, we would expect the average size to be 9+5+5 =19. Empirically, we see a mean envelope width of ~22, not far off this estimate. For two average reads of 10kb and ~90,000 signals, this envelope would constrain the search to just 0.024% of the entire matrix, an efficiency which helps justify our use of a pairwise sequence alignment step.*

For reproducibility, the authors should specify which commit of the software was used for the analysis here or tag a release and specify the tag.

Software issues (as of commit 00ed544):

- Running "poreover train --data poreover/examples/training/toy --training_steps 100" throws "poreover: error: unrecognized arguments: --training_steps 100". I am guessing at some point this argument was changed to epochs, but this should be updated.

- poreover/examples/training/toy is not a directory but rather poreover/examples/training contains two gzipped files, which does not seem to be the correct input.

- Running "poreover call --fast5 poreover/examples/read.fast5 --model run-0 --out test.fasta" throws an error as expected because the model training failed, but the error thrown is "poreover call: error: the following arguments are required: --weights" so it does not look like this command is providing a required argument.

*Thank you for pointing this out. The README file has been corrected and expanded to include a more detailed tutorial. If this manuscript is accepted, we will also deposit this version of the source code in a DOI-assigning repository, as stipulated by the journal.*

Reviewer #3: The author developed an algorithm and a package aimed at improving the accuracy of nanopore sequencing methods when making use of the 1D2 protocol, protocol that sequence both strands of a DNA duplex sequentially. The originality of the method is the use on an intermediary results of available basecallers : the probability over the possible bases assigned at each current step for both complementary sequences. By using this information, through the use of a beam search algorithm on both strands, the authors manage to improve the accuracy of the last basecaller from nanopore, bonito, from an accuracy of 93.3 to 97.7 %

Major critique :

1) No clear superiority of their algorithm was demonstrated. A possible comparison could have been

to compare the present algorithm (using guppy output probability) to the 1D2 algorithm in guppy.

*We address this issue in more detail in response to a comment from Reviewer #1. While we have not been able to successfully apply pair decoding to probabilities from Guppy, this is likely due to the idiosyncratic nature of its "flip-flop" CTC model, which differs from all the other basecallers tested.*

*To support the generality of our method to basecallers that use the standard form of CTC, we have added an additional pair decoding benchmark using the recent community basecaller DeepNano-blitz.*

*As consensus approaches should be improved by more accurate sequences as input, a possible concern might be comparing consensus accuracy between more and less accurate basecallers. In our case, however, the Bonito and Guppy basecalling networks show similar 1D accuracies, so the use of the Bonito basecaller is not providing an unfair advantage to our algorithm.*

*Ultimately, while a relative decrease in the error rate is desirable, the final absolute accuracy is generally the most biologically relevant metric. Our consensus approach both yields larger decreases in relative error rate compared with Guppy, and when combined with Bonito, also yields the highest absolute 1D2 accuracy.*

2) Two month ago pair decoding was added to bonito basecaller. A comparison between poreover and bonito should be run. Without it, it is very difficult to assess the added value of the current method.

*It is understandable that the reviewer would request this comparison, under the circumstances, but to clarify: the Bonito pair decoding method is an implementation of the 2D beam search we present here. After the release of this manuscript in preprint form earlier this year, we were contacted by the Bonito team, who sought to replicate our results.*

3) Before a release of the package, a detailed step by step method explicitly showing how to use

poreover should be added, as for now, by reading the documentation it is not explained how to

generate examples/1d2/read1.npy and examples/1d2/read2.npy which are

required to use poreover in pair-decoding. Also it is mentioned that it is necessary to recompile

bonito to use the method developed by the authors, but there is no information about this on the

readme.

*Thank you for pointing this out. The README file has been expanded to include a step-by-step tutorial for installing a modified Bonito and running pair decoding.*

Minor :

3) The author should mention the running time of their algorithm, as nanopore sequencers generate large volume of data, and this information could be useful in the decision to use or not the current algorithm.

*Thank you for pointing this out. A note on running times has been added to the Supplementary Information.*

4) What is the effect of the bandwidth W on the final accuracy?

*Our program uses a default beam width of 5. Beam sizes of 10 and 25 were tested with negligible increases in accuracy.*

5) A more symmetric approach could have been use in the pair decoding algorithm: Here, if I

understood correctly, at t1 the first strand is used to select the top W sequences , and the second strand is used, at t1, from t2=0 to T to improve the accuracy to the global forward probability. I call this probability P(0→t1,t2=0→T2). A more symmetric approach could have been used with the exact same algorithm but t1 and t2 are increased from one at each time, and the top W sequence on both P(0→t1,t2=0→T2) and P(t1=0→T1,0→t2) are selected.

*We thank the reviewer for this illuminating suggestion. If we understand correctly, this is equivalent to iterating over one row and one column of at the same time, with each sequence in the beam being scored as the max probability of read 1 in the that column times the maximum probability of read 2 in the row. We have implemented this algorithm in PoreOver and tested it. It yields results that are quite comparable to our initial algorithm, but is faster when adapted to run on our alignment envelopes. We have repeated the benchmarks in the manuscript using this variation and changed it to be the default option in PoreOver.*

**Second round of review**

**Reviewer 2**

I believe the authors have sufficiently addressed my comments. I recommend publication and congratulate the authors on a very nice piece of work. My only remaining request is that the authors add a few lines, either in the main text or the SI, explaining the <2% failure rate and mean envelope width as they have done in the response to reviewers.
